# Seismic data of a rockslide: Evaluation of noise levels, site effects, frequency content and identification of seismic phases

**DOI:** 10.1016/j.dib.2020.105250

**Published:** 2020-02-07

**Authors:** Mar Tapia, Marta Guinau, Pere Roig, Cristina Pérez-Guillén, Emma Suriñach, Giorgi Khazaradze

**Affiliations:** a Group RISKNAT, Institut Geomodels, Universitat de Barcelona, Faculty of Earth Sciences, Department of Earth and Ocean Dynamics, Barcelona, Spain; b Laboratori d’Estudis Geofísics Eduard Fontserè, Institut d’Estudis Catalans (LEGEF-IEC), Barcelona, Spain

**Keywords:** Seismic data, Translational rock slide, Seismic quality control, Waves identification, Site effects

## Abstract

Seismic data can provide information to deduce the occurrence of mass movement events, their release time, event location and dynamics characterization [1]. Nevertheless, the effect of local site amplifications, the level of seismic noise and the frequency content of the signals are important constraints to correctly identify and describe these types of events. In this article we provide data on: site effects, power spectral densities, polarization particle motion and spectrograms generated by a rockslide (∼450 m^3^) (hereinafter NR) recorded in two permanent seismic stations (EPOB and POBL) located ∼10 km from the source. Original data are available through the International Federation of Digital Seismograph Networks (FDSN, http://www.fdsn.org) for POBL and on request from Instituto Geográfico Nacional (IGN, http://www.ign.es) for EPOB. POBL and EPOB site effects analysis by means of Horizontal-to-Vertical spectral ratio (H/V) technique shows important signatures in POBL signal between 1 and 10 Hz, indicating strong amplification effects at these frequencies, not present in EPOB. For frequencies >1 Hz, Power Spectral Densities (PSD) are higher in POBL than in EPOB, indicating that POBL is noisier than EPOB. Based on the H/V and PSD analyzes, the EPOB station data was deemed preferable over the POBL, to conduct the research presented in the related article [1]. Particle polarization motion data enabled the identification of the arrivals of P, S, and superficial waves, confirming that P_g_ waves were correctly identified, providing necessary information for the event location in the research article [1]. Moreover, EPOB and POBL spectrograms together with the Fourier transform are included to analyze their content in the frequency domain showing that the expected high frequency phenomenon of the rockslide recorded at 10 km is attenuated and only the low frequency content between 1 and 15 Hz is recorded.

**Table:** Specifications Table

Subject area	*Physics, Earth Sciences, Remote Sensing*
More specific subject area	*Geophysics, seismology*
Type of data	*Seismic data and Images (HV, PSD curves, particle motion and spectrograms plots) and text file.*
How data was acquired	*Permanent seismic stations with 3-component broadband seismometers (Guralp CMG3T and Geotech KS2000M) together with three channel digitizers, GPS and data transmission systems respectively.*
Data format	*Raw, processed and graph*
Experimental factors	*Raw data were corrected from baseline and trend, and band filtered with fourth order causal Butterworth filters, followed by a further data analysis.*
Experimental features	*Data were selected from continuous seismic data.*
Data source location	***POBL****(located at Monestir de Poblet, Tarragona, Spain):* *Lat: 41°22′45.24″N; Lon: 1° 5′0.86″E* ***EPOB****(Tarragona, Spain): Lat: 41°21′9.72″N Lon: 1° 5′0.86″E*
Data accessibility	Original raw data are available through the International Federation of Digital Seismograph Networks (FDSN, http://www.fdsn.org) for POBL or directly at http://ws.icgc.cat/fdsnws/dataselect/1/query?starttime=2013-05-05T08:41&endtime=2013-05-05T08:42&network=CA&station=POBL&location=*&channel=*&nodata=404 and on request to Instituto Geográfico Nacional (IGN, http://www.ign.es) for EPOB. Also the data in SAC format can be accessed through LEGEF server at http://sismic.iec.cat/POBL_EPOB_SACdata.zip
Related research article	Guinau et al. “Remote sensing and seismic data integration for the characterization of a rock slide and an artificially triggered rock fall” Engineering Geology, 257 (2019) 105,113 (https://doi.org/10.1016/j.enggeo.2019.04.010)

**Table:** 

**Value of the Data** • The data provide new insights on the identification of P, S and superficial waves from seismic signals produced by relatively small rockfall events (Volume< 500 m^3^) and recorded at relatively long source-receiver distances (>10 km). • The data can be used as a source of information to determine the occurrence time of rockfall events. • The data revealed the possibility to deduce the location of small rockfall events at long distances using the single station location method. • The data revealed that without a thorough knowledge of the geological characteristics of the source-receiver path, seismic source energies associated to small rockfalls cannot be reliably deduced from seismic signals with frequency content up to 15 Hz. This is especially true for large distances.

## 1 Data

The seismic data analyzed and presented here were recorded in two permanent 3-component broadband seismometers. These seismic stations recorded the rockslide event on May 5th, 2013 occurred in the village of La Riba (NE Spain). These two stations are EPOB, located at 9.5 km NW of La Riba site, belonging to the Spanish seismic network (Instituto Geográfico Nacional, IGN) and POBL, located at 10.7 km NW of it belonging to Laboratori d’Estudis Geofísics Eduard Fontserè, Institut d’Estudis Catalans (LEGEF-IEC), located less than 3 km from each other. (Fig. 6 in Ref. [1]). Both stations are deployed mainly for earthquake monitoring in their respective regions. The EPOB and POBL stations continuously record seismic data at a sampling rate of 100 Hz and 50 Hz, respectively. They use 3 channel 24-bit digitizers with a flat response between 0.01 and 50 Hz (EPOB) and 0.01–25 Hz (POBL). Original data are available through the International Federation of Digital Seismograph Networks under the tutelage of IRIS (Incorporated Research Institutions for Seismology) as an international public seismic data repository (FDSN, http://www.fdsn.org) for POBL and on request to IGN (http://www.ign.es) for EPOB. The original format of this seismic data is mseed format, one of the international seismic data format, among others as can be SAC format. Also these data in SAC format can be accessed through LEGEF server at http://sismic.iec.cat/POBL_EPOB_SACdata.zip. There are format converters to other seismic formats and ascii format available through the net.

**Fig. 1:**
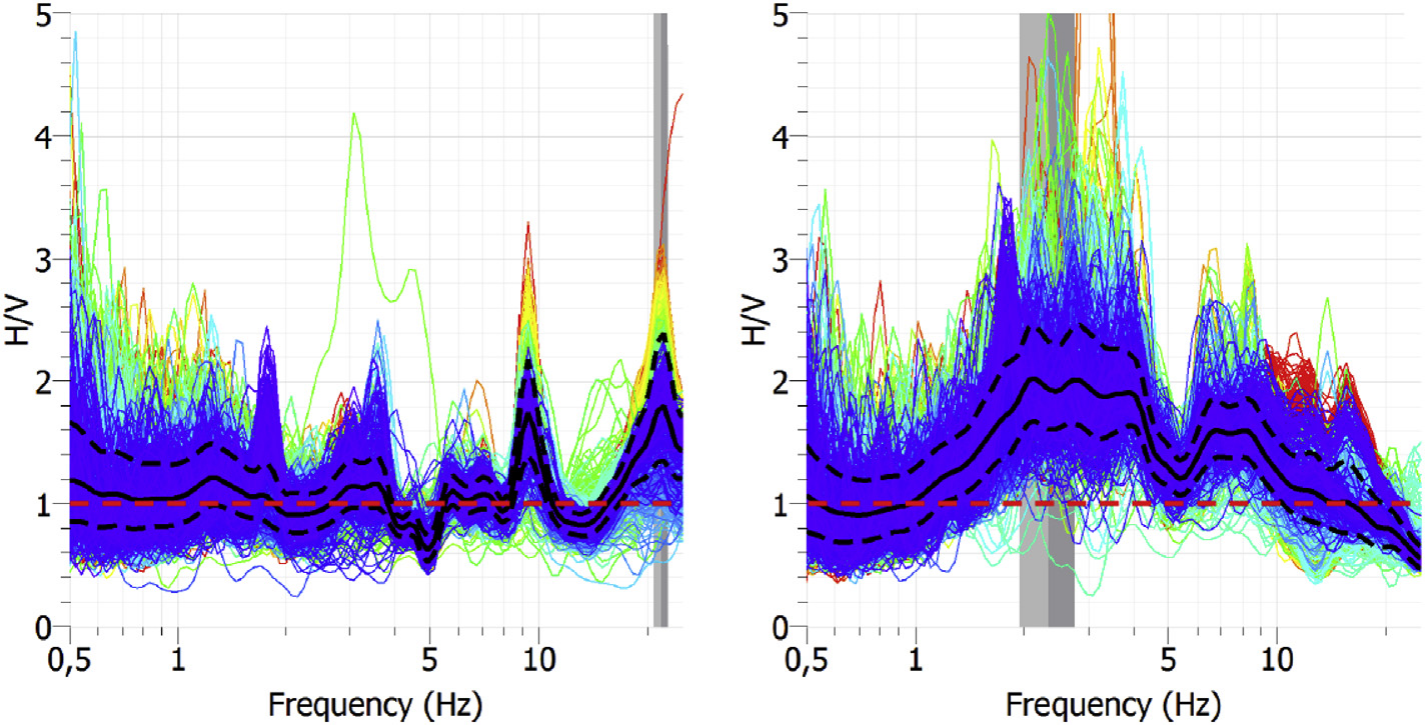
H/V average curves (solid black lines) with their standard deviations (black dashed lines) for EPOB (left) and POBL (right). One-day seismic noise data, with H/V curves from selected windows between 25 and 50 s. Grey vertical bands identify the obtained predominant frequency and standard deviation. Red horizontal line is added to ease the identification of the H/V = 1 ratio.

**Fig. 2:**
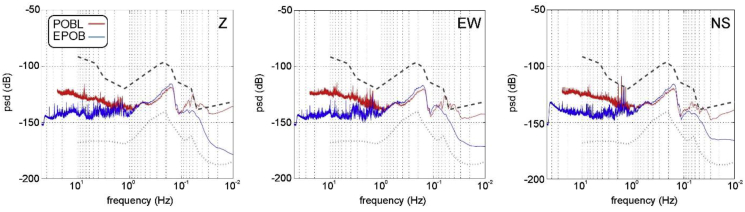
Z (left), EW (central) and NS (right) PSD curves for POBL (red line) and EPOB (blue line) for 1-day data combined with LNM and HNM curves (dashed lines).

**Fig. 3:**
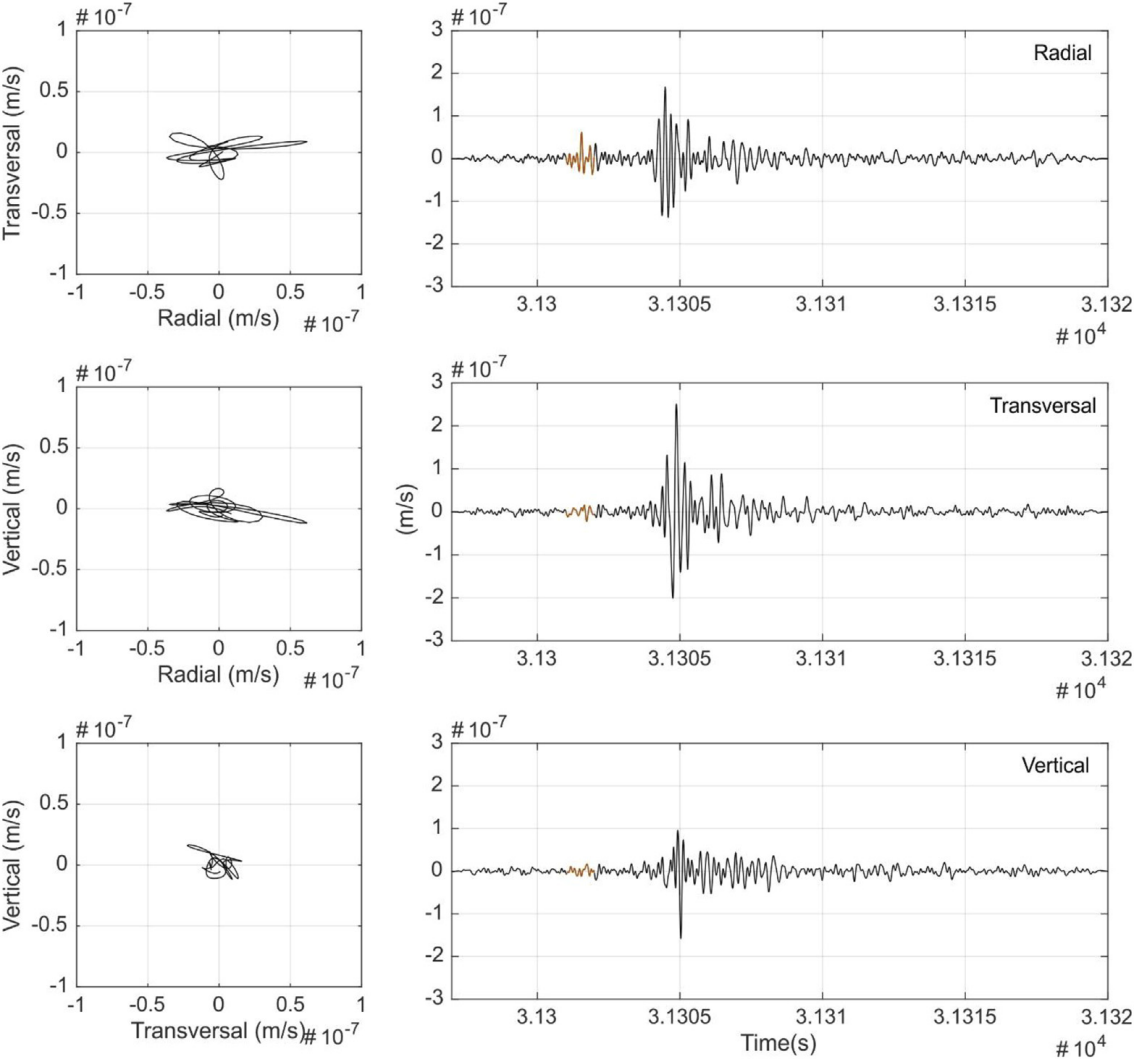
P-waves particle motion data. Left: Transverse-radial (TR), vertical-radial (VR) and vertical-transverse (VT) particle motion plots. Right: Seismic time signal 1–9 Hz filtered and the signal part used for the particle motion (orange).

**Fig. 4:**
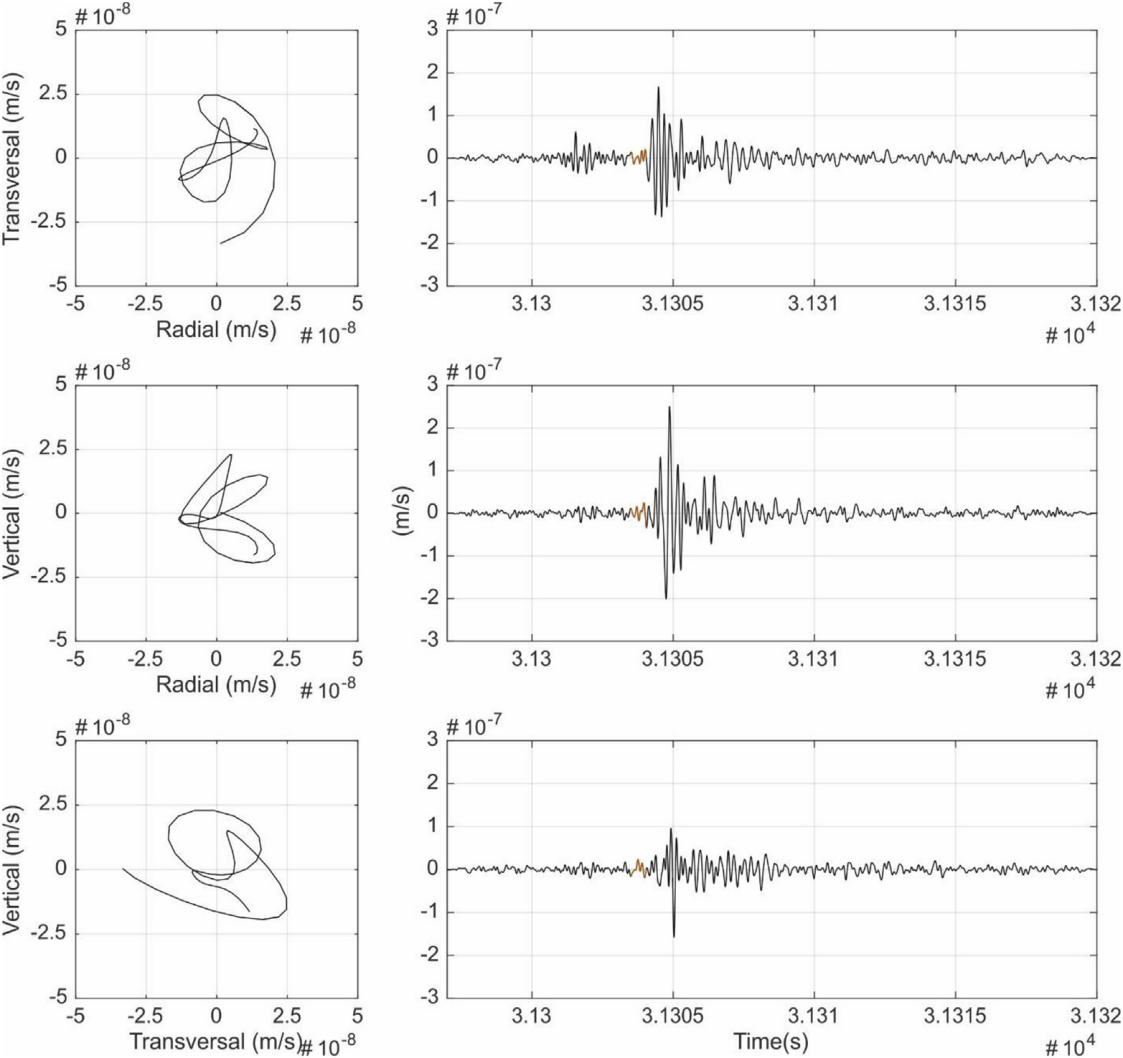
S-waves particle motion analysis. Left: Transverse-radial (TR), vertical-radial (VR) and vertical-transverse (VT) particle motion plots. Right: Seismic time signal 1–9 Hz filtered and the signal part used for the particle motion (orange).

**Fig. 5:**
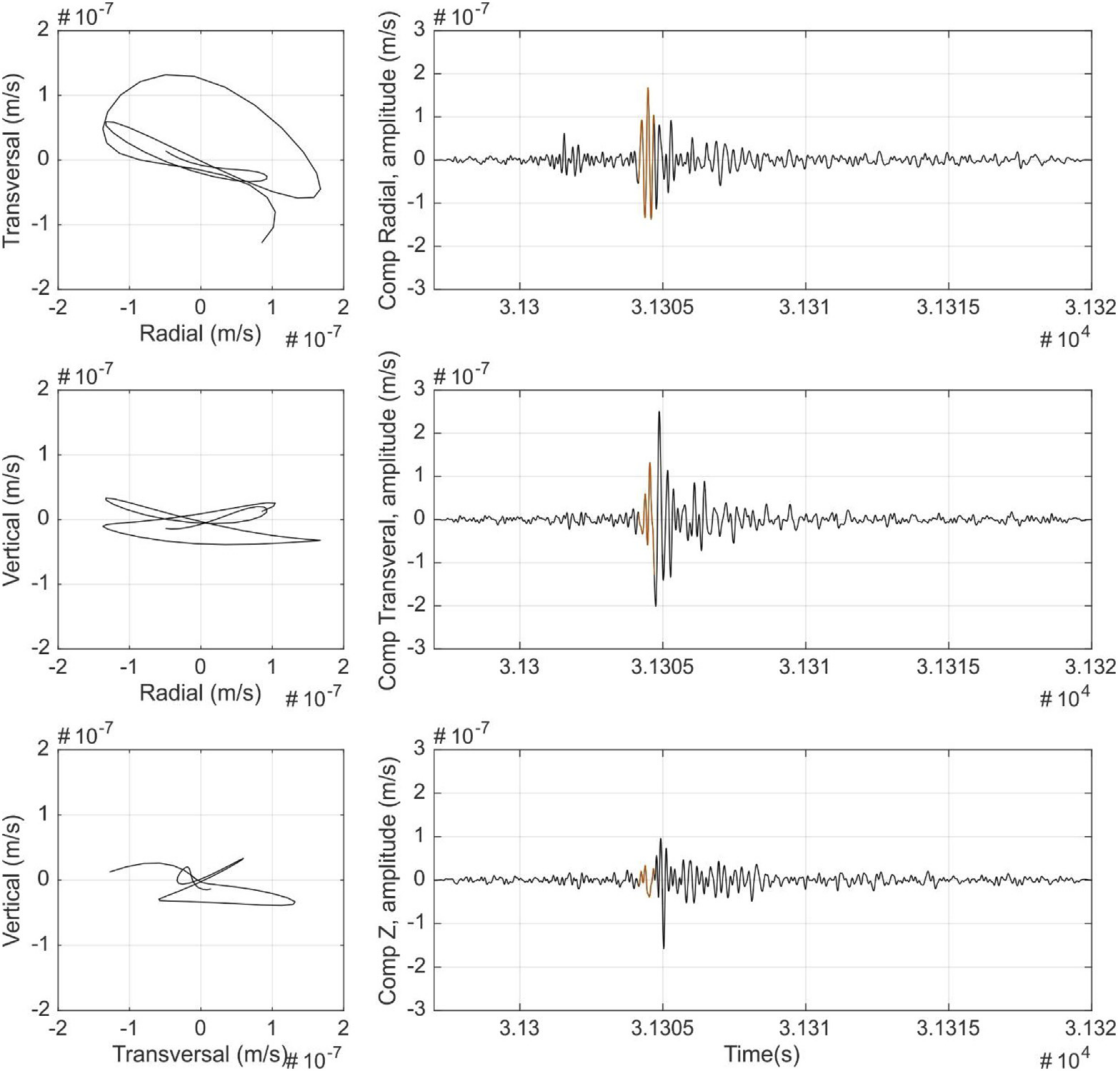
Love waves particle motion analysis. Left: Transverse-radial (TR), vertical-radial (VR) and vertical-transverse (VT) particle motion plots. Right: Seismic time signal 1–9 Hz filtered and the signal part used for the particle motion (orange).

**Fig. 6:**
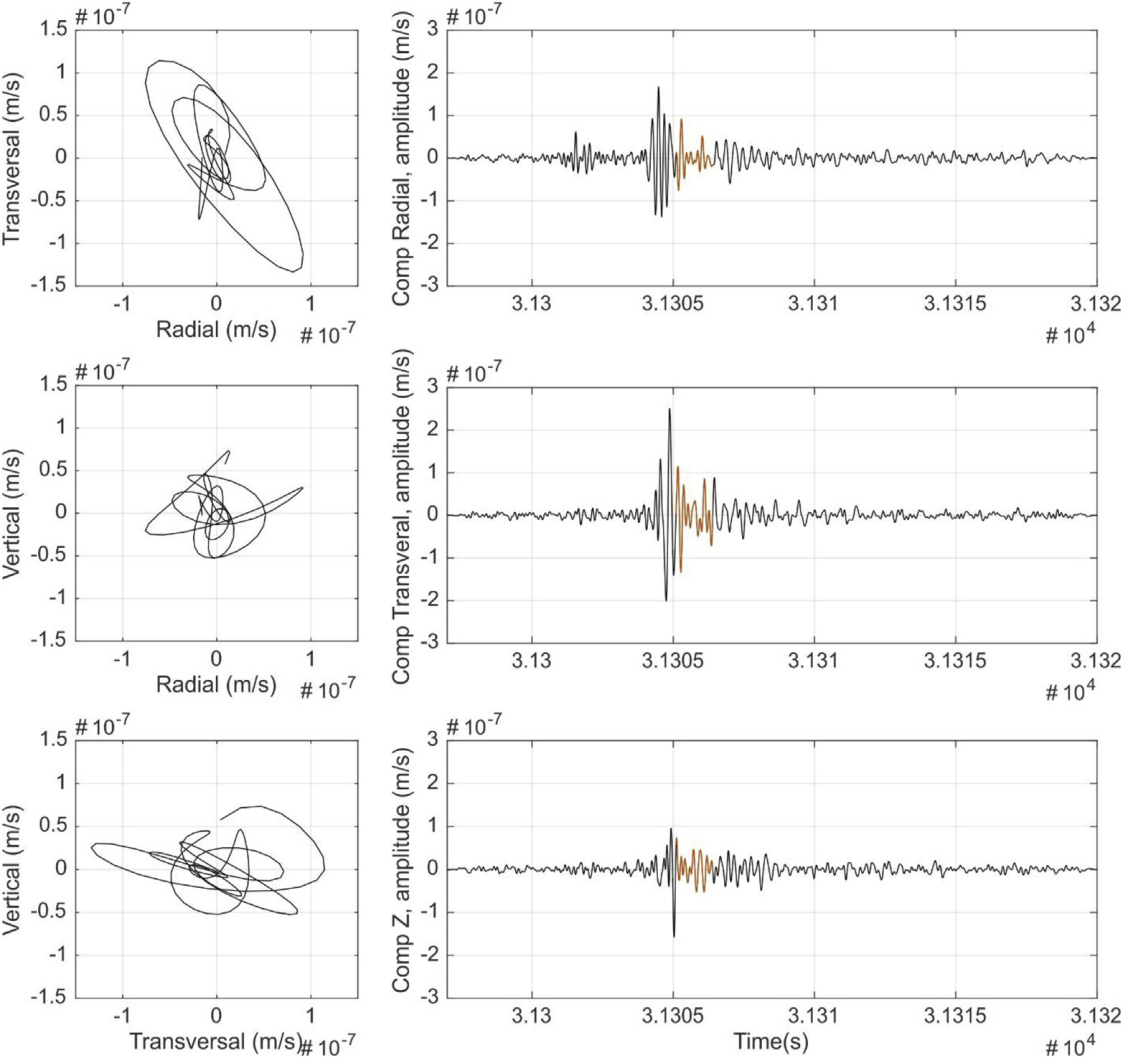
Rayleigh waves particle motion analysis. Left: Transverse-radial, vertical-radial and vertical-transverse particle motion plots. Right: Seismic time signal 1–9 Hz filtered and the signal part used for the particle motion (orange).

**Fig. 7:**
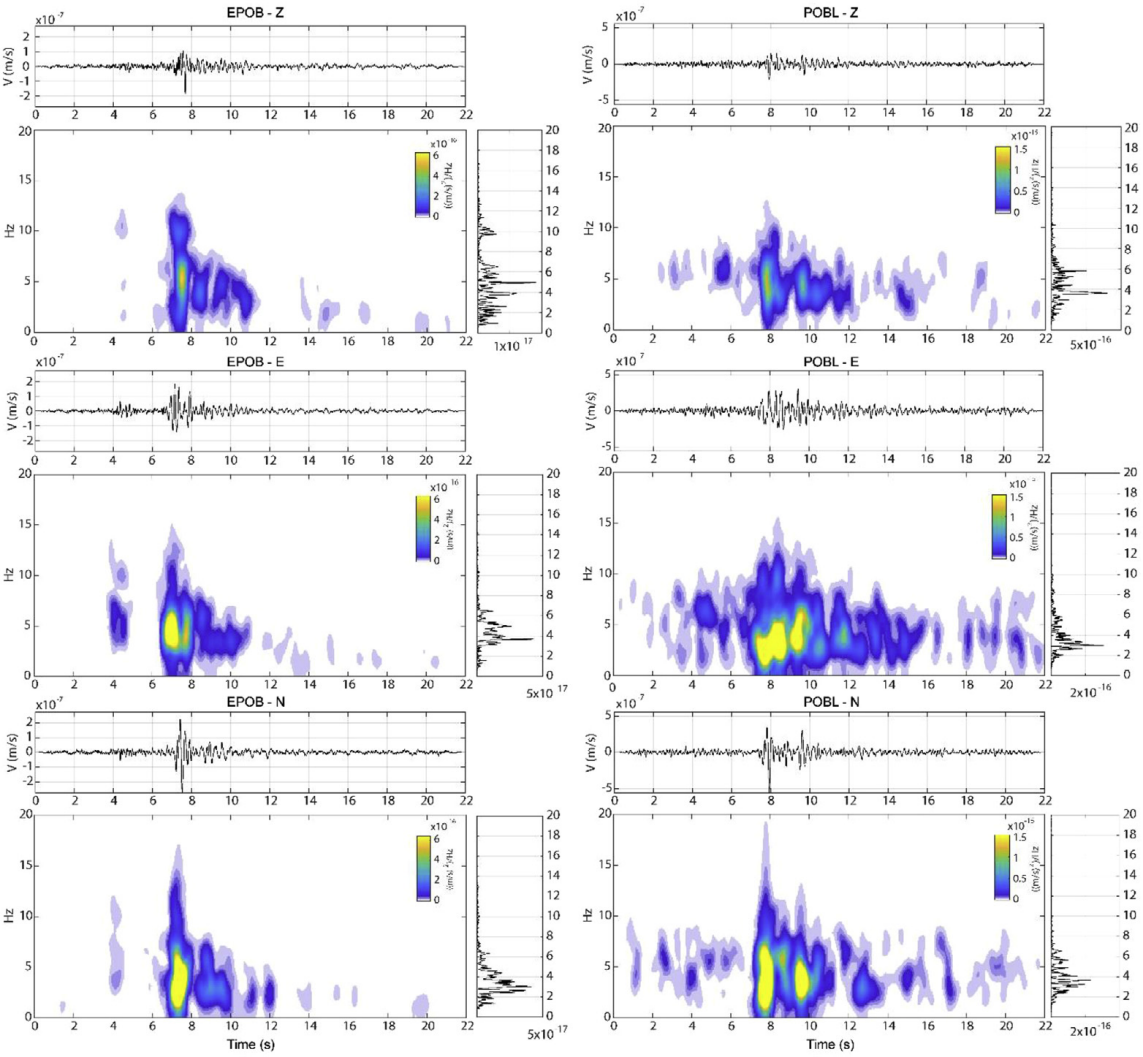
Z, EW and NS spectrogram of 1 s windows overlapping 90% for EPOB (left) and POBL (right) together with the Fourier transform amplitude and temporal record. Seismic signals are 1 Hz filtered by a fourth order Butterworth high pass filter.

Fig. 1 shows the result of a Nakamura's H/V analysis [2] of the seismic data to show site effect at each station. Fig. 2 shows the result of the Power Spectral Densities computation from the seismic data to show background seismic noise from each station. From Fig. 3, Fig. 4, Fig. 5, Fig. 6 a particle motion analysis is presented to identify the different seismic waves involved in the recorded rockslide. Fig. 7 shows a time-frequency analysis of the seismic data to the characterization of the recorded rockslide.

## 2 Experimental design, materials and methods

### 2.1 Site effects for POBL and EPOB seismic stations

Site effects at EPOB and POBL sites were evaluated using Nakamura's H/V method [2] as a simple seismic noise (microtremors) measurement technique for estimating amplification of ground motions. Nakamura's H/V method identifies predominant frequencies where seismic wave amplifications occur [[2], [3]].

Fig. 1 shows the results of applying the H/V method using Geopsy software [4] to EPOB and POBL microseismic data. EPOB shows some peaks around 10 and 20 Hz but they are not very prominent, oscillating around 1 H/V ratio, indicating no remarkable amplification effects in this frequency range. On the contrary, the POBL site shows important signature between 1 and 10 Hz above 1 H/V ratio, which indicate amplification effects at these frequencies.

### 2.2 POBL and EPOB seismic stations noise analysis

The calculation of the Power Spectral Densities (PSD) for both, EPOB and POBL stations, was performed for a winter day, which is the noisiest period of the year. At frequencies between 0.1 and 1 Hz that correspond to seismic noise from oceanic oscillations, EPOB and POBL PSDs are similar (Fig. 2). For frequencies greater than 1 Hz, POBL always shows higher PSD values than EPOB, indicating that the POBL station is noisier than EPOB.

In order to compare the seismic noise characteristics of the EPOB, POBL and the reference models, Fig. 2 shows the seismic noise characteristics in EPOB and POBL sites, together with the curves of low noise (LNM) and high noise (HNM) reference models [[5], [6]].

### 2.3 EPOB particle motion of phases for NR event

In order to check the seismic phases identified in EPOB and POBL seismic signals produced by the rockslide event, a classical particle motion analysis was done using EPOB data. A fourth order Butterworth band pass filter was applied between 1 and 9 Hz and the NS and EW components were rotated to radial (R) and transverse (T) components using an angle of 116°, which is the azimuth of the event deduced in section 6 of the associated research article [1].

Fig. 3, Fig. 4, Fig. 5, Fig. 6 show the results of the particle motion analysis from different time interval (marked in the waveforms of each figure) for radial-transverse, radial-vertical and transverse-vertical planes.

Fig. 3 corresponds to the part of the event identified as P waves. These P waves are predominant, as expected, in the radial R component and almost disappear in the transverse T component, due to their longitudinal elastic body wave nature. On the contrary, as would be expected, S wave analysis in Fig. 4, show mainly the presence of transverse T component (vertical-transverse plane), although some minor part in the radial R component can also be identified. This might be caused by noise contamination due to the low amplitudes or other reflected phases. Love waves (Fig. 5), also as expected, mainly can be seen as a transverse horizontal motion, with a lower signature in a vertical component than in the radial-transverse RT plane. Rayleigh wave analysis (Fig. 6) shows motion in the direction of propagation (radial R component) and perpendicular (in a vertical plane) and elliptical motion due to its phased motion.

### 2.4 EPOB frequency content for NR event

A time-frequency analysis of the POBL and EPOB seismic data was used for selecting suitable filters in the seismic analysis used throughout the research article [1].

Fig. 7 shows the time-frequency data for the POBL and EPOB seismic data, showing energy up to 15 Hz with relevant values around 5 Hz. This shows the effect of wave attenuation with distance for a rock slide that is expected to have high frequency content. As expected, POBL is also characterized by a noisier signal for the same frequency range (see Section 6.1 of the related research article [1]).
